# Choroidal Optical Coherence Tomography Angiography: Noninvasive Choroidal Vessel Analysis via Deep Learning

**DOI:** 10.34133/hds.0170

**Published:** 2024-09-10

**Authors:** Lei Zhu, Junmeng Li, Yicheng Hu, Ruilin Zhu, Shuang Zeng, Pei Rong, Yadi Zhang, Xiaopeng Gu, Yuwei Wang, Zhiyue Zhang, Liu Yang, Qiushi Ren, Yanye Lu

**Affiliations:** ^1^ Institute of Medical Technology, Peking University Health Science Center, Peking University, Beijing 100191, China.; ^2^ Department of Biomedical Engineering, Peking University, Beijing 100871, China.; ^3^ Department of Ophthalmology, Peking University First Hospital, Beijing 100034, China.; ^4^ National Biomedical Imaging Center, Peking University, Beijing 100871, China.

## Abstract

**Background:** The choroid is the most vascularized structure in the human eye, associated with numerous retinal and choroidal diseases. However, the vessel distribution of choroidal sublayers has yet to be effectively explored due to the lack of suitable tools for visualization and analysis. **Methods:** In this paper, we present a novel choroidal angiography strategy to more effectively evaluate vessels within choroidal sublayers in the clinic. Our approach utilizes a segmentation model to extract choroidal vessels from OCT B-scans layer by layer. Furthermore, we ensure that the model, trained on B-scans with high choroidal quality, can proficiently handle the low-quality B-scans commonly collected in clinical practice for reconstruction vessel distributions. By treating this process as a cross-domain segmentation task, we propose an ensemble discriminative mean teacher structure to address the specificities inherent in this cross-domain segmentation process. The proposed structure can select representative samples with minimal label noise for self-training and enhance the adaptation strength of adversarial training. **Results:** Experiments demonstrate the effectiveness of the proposed structure, achieving a dice score of 77.28 for choroidal vessel segmentation. This validates our strategy to provide satisfactory choroidal angiography noninvasively, supportting the analysis of choroidal vessel distribution for paitients with choroidal diseases. We observed that patients with central serous chorioretinopathy have evidently (*P* < 0.05) lower vascular indexes at all choroidal sublayers than healthy individuals, especially in the region beyond central fovea of macula (larger than 6 mm). **Conclusions:** We release the code and training set of the proposed method as the first noninvasive mechnism to assist clinical application for the analysis of choroidal vessels.

## Introduction

**Fig. 1.**
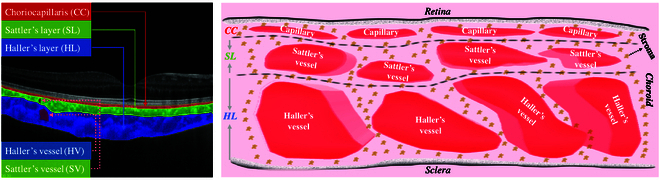
The structure of choroidal tissues, including CC, SL, HL, HV, and SV.

The choroid, serving as the primary vascular layer of the human eye, plays a crucial role in supplying oxygen and nourishment to the outer retina. As illustrated in Fig. 1, the choroidal structure can be subdivided into 3 sublayers based on the vessel distribution: choriocapillaris (CC), Sattler’s layer (SL), and Haller’s layer (HL) [1]. These sublayers are vascular beds that contain the capillary, the middle-sized Sattler’s vessel (SV), and the large-sized Haller’s vessel (HV), respectively. As the most vascularized structure, choroidal biomarkers have reflected associations with numerous retinal and choroidal diseases, including age-related macular degeneration [2], uveitis [3], Vogt–Koyanagi–Harada syndrome (VKH) [4], and central serous chorioretinopathy (CSC) [5–8]. While choroidal vessels (CVs) significantly impact ocular health, the specific role of vessels in choroidal sublayers, especially SV and HV, remains unexplored due to the lack of angiography mechanisms capable of visualizing and evaluating them.

**Fig. 2.**
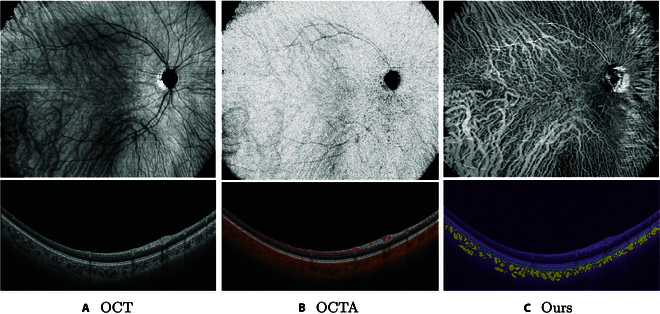
(A) OCT choroidal en face (top) and B-scan (down). (B) OCTA choroidal en face (top) and B-scan (down). (C) Our en face (top) and B-scan (down). Our method can clearly catch the deep CVs with less artifact by other tissues such as retinal vessels and choroidal stroma.

In clinical practice, the invasive imaging method, indocyanine green angiography (ICGA) [9], is considered the gold standard for visualizing CVs. However, ICGA lacks the ability to provide volumetric information, failing to resolve the vessels of different choroid sublayers. Compared with ICGA, the dense collection of optical coherence tomography (OCT) B-scans allows for the precise localization of choroidal layers, facilitating the generation of a choroidal en face projection for visualizing choroidal structures. Unfortunately, current OCT angiography (OCTA) [10] faces challenges in gathering sufficient photons of deep CVs (HV&SV), caused by the rapid flow of CC [11,12] as well as the presence of melanin particles located within the retinal pigment epithelium (RPE) [13,14]. As illustrated in Fig. 2B, the OCTA presents dark at the location of HV and SV, confining its capability of evaluating CVs in clinical practice. Consequently, there is still an urgent need for a method to noninvasively capture 3-dimensional (3D) choroidal vascular information.

To fill this gap in the choroidal analysis, our work proposes to segment SV&HV on noninvasively collected OCT B-scans with deep learning to comprehensively reconstruct the CV distributions. However, the reconstruction process requires densely collecting a cube of B-scans (1,024 slices) in a few seconds, resulting in low choroidal quality, substantial speckle noise, and blurry tissue boundaries. Thus, existing vessel segmentation methods, which focus only on high-definition (HD) B-scans collected through meticulous focal point adjustments and multiple overlapped averages (more than 30 times) for accurate annotation, encounter challenges in adapting to densely collected OCT B-scans. To tackle these challenges, we further formulate the choroidal angiography process mentioned above as cross-domain OCT segmentation and elaborate on a novel deep learning framework. The proposed framework ensures that the SV and HV segmentation model, initially trained on HD B-scans, can also effectively perform on low-quality B-scans, thus saving human resources for both annotation during training and reconstruction during inference.

In the proposed framework, the HD B-scans serve as the source samples with available labels, enabling the segmentation model to learn in a supervised manner. Simultaneously, B-scans that are densely collected within a few seconds are used by a target branch to perceive their distribution without accessing their annotations. Additionally, the framework includes an adaptation branch to reduce the distribution shift of the choroidal structure between the 2 types of B-scans. This proposed framework proves to be a valuable and efficient tool for conducting 3D analyses of CV distribution. Notably, with the help of this tool, our investigations reveal a significant (*P* < 0.05) reduction in vascular index at all choroidal layers among CSC patients compared to healthy individuals.

In a nutshell, our main contributions are 3-fold:

• Our work proposes the first clinical tool enabling 3D evaluation of choroidal sublayer vessels solely based on noninvasive imaging.

• We elaborate a deep learning framework that adopts self-training and adversarial learning strategies to accommodate the different types of B-scans used for annotation (training) and reconstruction (inference) processes.

• Our experiments demonstrate the effectiveness of the proposed framework and its clinical potential in choroidal analysis, revealing a significant reduction in the vascular index of CSC patients

## Methods

### Preliminaries

#### CNN-based choroidal structure extraction

Choroidal structure extraction focuses on extracting the choroidal layer or CVs from OCT B-scans. Specifically, Sui et al. [15] and He et al. [16] addressed choroidal layer segmentation in 2 stages: First, features are extracted by a convolutional neural network [17], and then a graph search method is utilized to locate the choroidal surfaces. Chen et al. [18] incorporated enhanced depth imaging OCT into a 3D graph search to segment choroidal layers. Zhang et al. [19] simplified this 2-stage process with semantic segmentation to segment the choroidal layer and attempted to eliminate the retinal vessel shadow for better visualization of the choroidal structure with OCT en face. Li et al. [20] further explored adopting a 3D residual U-Net for choroidal layer segmentation. Recently, some works have also been proposed to tackle the more challenging task of CV segmentation [21]. Liu et al. [22] annotated 40 swept-source optical coherence tomography B-scans, each of which has been averaged 32 times to enhance choroid quality. These high-quality B-scans were used to train a segmentation model to segment CVs. Zhu et al. [23] adopted a multi-task learning strategy to design a segmentation model that synergistically segments choroidal layers and vessels on B-scans averaged 30 times for enhanced depth imaging optical coherence tomography. Huang et al. [24] proposed a 3D convolutional neural network (CNN)-based method that considers neighboring B-scans when segmenting CVs. Unlike these methods, our work is the first to focus on better extracting vessels of choroidal sublayers (HV and SV) from low-quality B-scans (averaging only 2 times) with a large coverage range (12 mm × 12 mm), which is more challenging and aligns with clinical requirements.

#### Semisupervised learning

Semisupervised learning (SSL) explores training neural networks with both labeled and unlabeled images, where unlabeled images are sampled from the same distribution as labeled images. Some SSL methods first generate hard annotations for the unlabeled data and combine them with labeled images to jointly train the model. For example, Lee [25] indicated that simply using the maximum predicted probability of the model trained on labeled data to annotate unlabeled data for an additional stage of training can already boost performance. Chen et al. [26] trained 2 models with different initializations on labeled data and used their predictions to supervise each other. Different from these approaches, Miyato et al. [27] focused on the consistency between an image and its perturbation, proposing the virtual adversarial training strategy that approximates the perturbation inspired by adversarial attacks. Ouali et al. [28] further designed the cross-consistency training strategy to ensure the invariance of predictions across multiple perturbations, including adding noise, spatial dropout, and virtual adversarial training. Tarvainen and Valpola [29] proposed the mean teacher strategy, which uses an additional teacher network updated with exponential moving average (EMA) to supervise the student with consistency regularization. Sohn et al. [30] adopted 2 different types of augmentation strategies and incorporated self-training into the mean teacher framework to generate hard annotations. However, in our case, the unlabeled images have a different distribution, which can cause SSL methods to underperform. Therefore, unlike these methods, our work also focuses on how to effectively select representative samples from the unlabeled images to better account for the distribution shift in our case.

#### Cross-domain segmentation

Cross-domain image segmentation aims to train neural networks to account for discrepancies between training (source) and testing (target) samples. The adversarial learning strategy is commonly used in domain adaptation (DA) segmentation, forcing the learned features to confuse a domain discriminator [31–35]. Specifically, Tsai et al. [34] proposed a multi-level adversarial learning scheme that adds domain discriminators for both feature and output spaces. Based on this strategy, Liu et al. [32] further adopted SSL to utilize unlabeled target samples for the adaptation process. In addition to the domain discriminator, Hung et al. [36] and Spadotto et al. [33] engaged a generative adversarial network (GAN)-based SSL strategy and designed a multiple domain discriminator adaptation strategy, where an additional discriminator discerns ground truth from predictions. Tranheden et al. [37] explored an augmentation strategy that mixed target objects into the source background to create cross-mix samples for enhancing adaptation performance. However, in our case, the domain discrepancy is caused by both structural shifts and quality differences, making the domain discriminator tend to catch speckle noise to discern domain cues. Therefore, unlike these methods, our work also explores how to train the domain discriminator to capture more content-related cues.

### Data preparation for deep learning

The training data for our study were collected using 2 OCT devices with choroidal-visible modalities: the enhanced depth imaging optical coherence tomography device with a center wavelength of 870 nm (Spectralis; Heidelberg Engineering) and the swept-source device with a center wavelength of 1,050 nm (VG200D; CVI-Ssion Imaging). We randomly selected 88 HD B-scans from 92 groups of OCT B-scans, each with a resolution of 480 × 580 pixels, obtained from the eyes of children with myopia. These B-scans were meticulously scanned with well-designed focus point settings by expert optometrists and averaged 30 times to enhance image quality. Annotation of these B-scans was performed using the PAIR toolbox (http://www.aipair.com.cn/) by 3 experts trained according to established protocols [23,38]. Additionally, 768 low-quality B-scans were randomly selected from OCT cubes of 15 healthy subjects. These B-scans were initially scanned with standard focus points and averaged only 2 times, serving as unlabeled target samples for the training process. During the training process, weak augmentation was applied to all samples. This involved resizing the input B-scans to 512 × 512 pixels and then randomly cropping them to 448 × 448 pixels. For samples requiring strong augmentation (SA), additional techniques were applied to the weakly augmented B-scans, including Gaussian noise, color perturbation, Gaussian smoothing, and histogram shifting.

### Clinical data acquisition and preprocessing

We included 4 patients who had both paired golden standard ICGA and dense collected low-quality B-scan cubes to qualitatively evaluate the CV distribution. Additional low-quality B-scan cubes of 100 healthy subjects and 20 patients (comprising 17 CSC and 3 VKH patients) were also used to indicate our clinical potential. Those data were also collected by the swept-source device with a center wavelength of 1,050 nm (VG200D; CVI-Ssion Imaging).

### Noninvasive choroidal angiography framework

**Fig. 3.**
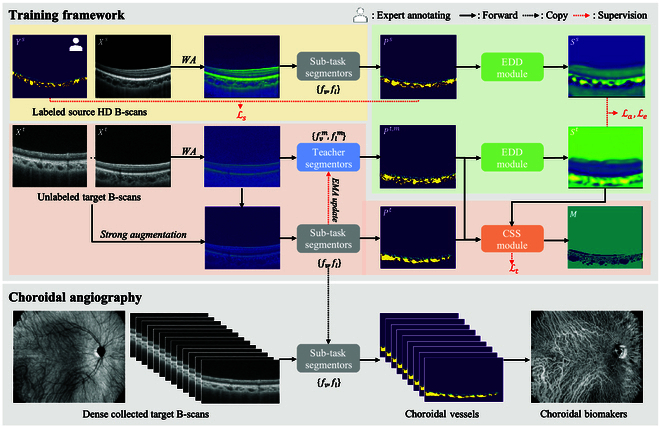
The mechanism of our proposed framework, containing the source branch (noted by yellow), target branch (noted by red), and adaptation branch (noted by green). In this framework, both labeled HD B-scans and unlabeled, densely collected low-quality B-scans are utilized to train the segmentors. The EDD module is trained to discriminative source segmentation and the ensemble target segmentation outputted teacher segmentor, avoiding the discriminator influenced by the different imaging quality between the 2 types of B-scans. The CSS module is adopted to select domain-confused samples to mask the target samples when calculating the self-training loss. After training, the subtask segmentor is used for choroidal antography.

To design an angiography strategy for evaluating vessels in choroidal sublayers, our approach focuses on adopting neural networks to automatically discern SV&HV from densely collected low-quality B-scans based on only annotations of HD B-scans. This process can be defined as a cross-domain segmentation task, i.e., training an SV&HV segmentation model with the consideration of the distribution shift between HD B-scan and original low-quality B-scans (Fig. 3).

Formally, given a cube of OCT B-scans **X** ∈ ℝ^*T*×*H*×*W*^ densely collected from a subject , its SV&HV distribution can be represented by a label volume:

$$Y_{t,h,w}=\left\{\begin{array}{c} 1,\;X_{t,h,w}\;\text{belongs to}\;\mathrm{SV}\\ 2,\;X_{t,h,w}\;\text{belongs to}\;\mathrm{HV}\\ 0,\;\text{otherwise} \end{array}\right.,$$ (1)

where *T* is the number of low-quality B-scans. *H* and *W* are the spatial resolution of each B-scan. Producing the predicted label volume can be seen as segmenting each low-quality B-scan of **X**:

$$P=\left\{P_{1},P_{2},\ldots ,P_{T}\right\}=\left\{f\left(X_{1}\right),f\left(X_{2}\right),\ldots ,f\left(X_{T}\right)\right\},$$ (2)

where *f*(·) is the segmentor, implemented by the neural network. **X***_t_* and **P***_t_* are the density and prediction score of the *t*_th_ B-scan, respectively. Based on **P**, the predicted label volume **Y**^∗^ can be computed by its maximum class prediction. For clarity, we use bold italic style to represent the corresponding tensors under slice level in the following, i.e., **X**, **P**, **Y**, and **Y**^∗^.

Since collecting low-quality B-scans is efficient and less expensive, it is feasible to collect a large amount of low-quality B-scans to form a target sample set to assist the above training process:

$$D^{t}=\left\{{\left(X^{t}\right)}_{i}\;|\;i<N^{t}\right\},$$ (3)

where *N^t^* is the number of the collected low-quality B-scans. $D^{t}$ represents the target sample set. Although the annotation of samples in $D^{t}$ is not available for the training process, we can still use those samples to align the feature distribution between HD B-scans and original low-quality B-scans. Moreover, those unlabeled target samples also support using self-training to further enhance SV&HV segmentation on low-quality B-scans.

Based on the above analysis, the training object of the segmentor *f*(·) can be formulated as follows:

$$L\left(D^{s},D^{t}\right)=L_{s}\left(D^{s}\right)+\lambda_{1}L_{t}\left(D^{t}\right)+\lambda_{2}L_{a}\left(D^{s},D^{t}\right)$$ (4)

where $D^{s}$ represent the source sample set. $L_{s}$ is the segmentation loss of source branch that supervises *f*(·) to discern SV&HV with the annotated source HD B-scans contained in $D^{s}$. $L_{t}$ is the self-training loss of target branch, which generates the pseudo-label to supervise the target low-quality B-scans in $D^{t}$. $L_{a}$ is the adaptation loss of adaptation branch that reduces the feature discrepancy between source and target samples. The structures of those 3 branches are detailed in the following sections.

### Source branch structure: Segmenting CVs

The source branch adopts source samples, i.e., HD B-scans, to train a segmentor in a supervised manner. Instead of implementing the segmentor as a multi-class semantic segmentation structure, our work decomposes this segmentation task into 2 subtasks, i.e., the vessels segmentation task and the choroid sublayer segmentation task:

$$Y^{s}=Y^{s,v}⊙Y^{s,l},$$ (5)

where ⊙ is the Hadamard product. **Y**^*s*,*v*^ ∈ {0, 1} represents the vessel map, and **Y**^*s*,*l*^ ∈ {0, 1, 2} represents the sublayer map. Compared with directly generating **Y***^s^*, this decomposition has 2 traits. On the one hand, **Y**^*s*, *l*^ gives empirical knowledge to distinguish ill-posed vessels caused by low imaging quality of original B-scan and choroidal disease. On the other hand, **Y**^*s*, *l*^ can provide the structure of choroidal sublayers, supporting the computation of more choroidal biomarkers, such as the thickness and vascular index [1,39,40].

Based on this decomposition, the segmentor *f*(·) is also disentangled into 2 subtask segmentors *f_l_*(·) and *f_v_*(·), which respectively generate choroidal sublayer map **P**^*s*,*l*^ and CV maps **P**^*s*,*v*^ for the final prediction:

$$P^{s}=P^{s,l}⊙P^{s,v}=f_{l}\left(X^{s}\right)⊙f_{v}\left(X^{s}\right),$$ (6)

where *f_v_*(·) and *f_l_*(·) are the subtask segmentors for vessel and sublayers, respectively. These segmentors can be implemented as UNet [41], AttUNet [42], SwinUNetR [43], etc. It is also possible to design the multi-task segmentor $\hat{f}(\cdot )=\left\{f_{v}(\cdot ),f_{l}(\cdot )\right\}$ with the multi-task mechanism [23] to train these 2 subtask segmentors in only one stage. For clarity, we adopt the multi-task segmentor to introduce our structure in the rest of this section and represent ${\hat{P}}^{s}=\left\{P^{s,l},P^{s,v}\right\}$, ${\hat{Y}}^{s}=\left\{Y^{s,l},Y^{s,v}\right\}$ (${\hat{P}}^{t}$, ${\hat{Y}}^{t}$ in the same way). Thus, the segmentation loss can be formulated as follows:

$$L_{s}\left(D^{s}\right)=\sum_{\left\{X_{i}^{s},Y_{i}^{s}\right\}\in D^{s}}‍l_{s}\left(\hat{f}\left(X_{i}^{s}\right),{\hat{Y}}_{i}^{s}\right),$$ (7)

where *l_s_*(·) is implemented using dice loss [44] to address the sample imbalance between background and choroidal tissues, benefiting from its consideration of both false positives and false negatives.

### Target branch structure: Utilizing unlabeled data

The target branch utilizes the unlabeled low-quality B-scan for self-training. Specifically, the unlabeled low-quality B-scan are first augmented with SA and then feed into the multi-task segmentor to generate the target predictions:

$$P^{t}=\hat{f}\left(g\left(X^{t}\right)\right)$$ (8)

where *g*(·) represents the operations of the SA [30].

Unlike the source samples, the target predictions do not have their corresponding ground truth to supervise the training process. Therefore, an additional teacher segmentor ${\hat{f}}^{m}(\cdot )$, having the same structure as the multi-task segmentor $\hat{f}(\cdot )$ and updated with its EMA [29], is adopted by target branch to generate the pseudo-annotations of **P***^t^*. In detail, this teacher segmentor can also produce a prediction of the unlabeled low-quality B-scan:

$${\hat{P}}^{t,m}={\hat{f}}^{m}\left(X^{t}\right).$$ (9)

Afterward, the class with the maximum predicted probability is used as the pseudo-annotation for these unlabeled target samples:

$${\hat{Y}}^{t,m}=\text{argmax}{\hat{P}}^{t,m}.$$ (10)

Considering the domain shift between the HD B-scan **X***^s^* and low-quality B-scan **X***^t^*, the pseudo-label ${\hat{Y}}^{t,m}$ suffers from large label noise. Thus, a confusion sample selector (CSS) module is designed for the target branch, which utilizes 2 gates to sample representable pixels for supervision:

$$\begin{array}{l} M^{c}=\left\{\begin{array}{ll} 1, & {\hat{P}}_{h,w}^{t,m}>\alpha \\ 0, & \text{otherwise} \end{array}\right. \end{array},\begin{array}{l} \;M^{d}=\left\{\begin{array}{ll} 1, & \beta_{2}>S_{h,w}^{t,m}>\beta_{1}\\ 0, & \text{otherwise} \end{array}\right. \end{array},$$ (11)

where *α*, *β*_1_, and *β*_2_ are the thresholds. ***M****^c^* represents the confidence gate that helps to select samples with confident teacher prediction (${\hat{P}}^{t,m}>\alpha$). ***M****^d^* represents the domain gate that focuses on selecting domain-confused samples based on the domain score $S_{h,w}^{t,m}$ generated by the EDD module (refer to the “Adaptation branch structure: Reducing domain shift” section). With the help of ***M****^d^*, the samples that are much like the source ($S_{h,w}^{t}<\beta_{1}$) are filtered out to avoid the segmentor overfitting the source distributions, i.e., only discerning vessels with sharpen boundaries as in HD B-scans. Meanwhile, the samples that satisfy $S_{h,w}^{t}>\beta_{2}$ are also filtered out due to their large distribution shift to the source, which will make them more likely to be misclassified by the source-trained teacher segmentor and causes label noise for self-training.

Based on these 2 gates, the proposed CSS module can select samples with confident prediction scores and confused domain distribution for the target branch, enhancing the self-training process of the multi-task segmentor with unlabeled low-quality B-scan.:

$$\begin{array}{ll} L_{t}\left(D^{t}\right)=\sum_{X_{i}^{s}\in D^{t}}‍ & l_{t}\left(M⊙P^{t},M⊙{\hat{Y}}^{t,m}\right),\\ & M=M^{d}⊙M^{c} \end{array}$$ (12)

where the loss term *l_t_*(·) is also implemented by dice loss [44].

### Adaptation branch structure: Reducing domain shift

The adaptation branch plays a crucial role in minimizing feature discrepancy between source and target samples through adversarial learning. At its core is a proposed EDD module *d*(·), which is implemented with 5 convolutional layers to discern the distribution of input samples:

$$S=\sigma \left(d\left(\hat{f}\left(X\right)\right)\right),$$ (13)

where ***S***_*h*,*w*_ → 0 means ***X***_*h*,*w*_ approaches to the source distribution, and *σ*(·) represents the sigmoid operation. The convolution layers of EDD were set by 4 × 4 kernel size, {64, 128, 256, 512, 1} channels, {2, 2, 1, 1, 1} strides, and {1, 1, 1, 1, 1} paddings. During the training stage of the EDD module, we compel it to distinguish the ensemble of the target output space from that of the source:

$$L_{e}=-\sum_{X^{t}\in D^{t}}‍\log(\sigma \left(d\left({\hat{P}}^{t,m}\right)\right)-\sum_{X^{s}\in D^{s}}‍\log(1-\sigma \left(d\left({\hat{P}}^{s}\right)\right).$$ (14)

${\hat{P}}^{t,m}$ is the prediction outputted by ${\hat{f}}^{m}(\cdot )$ with Eq. 9, which can be seen as a temporal ensembling of multi-task segmentor [29].

Based on the additional ensembling operation, our EDD module demonstrates superior adaptation to the quality difference between HD B-scans and low-quality B-scans compared to the discriminator of existing works [32–34]. Specifically, apart from the structure shift caused by the focus point settings, the domain shift in our cases is also rose from variations in imaging quality. The target sample, i.e., low-quality B-scans, contains significantly higher speckle noise [45] than the source sample, which has already been ensembled during the imaging process (averaged by multiple overlapped B-scans) [22,23]. This divergence in noise levels poses a challenge for discriminators, leading them to focus on the speckle noise rather than the choroidal structure when discerning the output distribution. Consequently, the training of the discriminator is easily trapped in local optimality. However, our EDD module incorporates a temporal ensembling step to simulate the imaging process of HD B-scan, which reduces the distribution gap caused by the quality shift and helps the discriminator better concern the factors of choroidal tissues.

In the training process of the multi-task segmentor, the trained EDD module is used to confuse $\hat{f}$(·) with adversarial learning, i.e., adopt reversed ground truth as supervision:

$$L_{a}=-\sum_{X^{t}\in D^{t}}‍\log(1-\sigma \left(d\left({\hat{P}}^{t,m}\right)\right)-\sum_{X^{s}\in D^{s}}‍\log(\sigma \left(d\left({\hat{P}}^{s}\right)\right).$$ (15)

It is worth to note that ${\hat{f}}^{m}$(·) is updated by EMA, making the first term of Eq. 15 not backward gradients on $\hat{f}$(·). Thus, $L_{a}$ can be simplified with only the second term of Eq. 15.

### Training process of deep learning model

The workflow for training the multi-task segmentor $\hat{f}$(·) is summarized in Algorithm 1. In each training iterator, we sample a batch of HD B-scans and low-quality B-scans from the source set $D^{s}$ and target set $D^{t}$, respectively. Subsequently, we augment the HD B-scans using weak augmentation and input them into the multi-task segmentor to generate the prediction score ${\hat{P}}^{s}$. This prediction is supervised by the annotation ${\hat{Y}}^{s}$ with $L_{s}$. For the unlabeled low-quality B-scan, we apply SA to perturb their distribution and feed them into the multi-task segmentor to generate their prediction ${\hat{P}}^{t}$. To obtain pseudo-annotations ${\hat{Y}}^{t,m}$ for supervision, the weak augmented low-quality B-scans are also fed into the teacher segmentor for prediction. These teacher predictions are then utilized by our EDD and CSS module to select the representative samples for self-training with $L_{t}$. Finally, based on the domain scores of ***S****^s^* and ***S****^t^* outputted by our EDD module, the DA loss $L_{a}$ is computed. This loss is combined with $L_{s}$ and $L_{t}$ to update the multi-task segmentor with stochastic gradient descent (SGD). Then, the teacher segmentor is updated with the EMA of corresponding parameters in the multi-task segmentor. Additionally, the EDD module is updated by SGD in the training iterators with $L_{e}$, computed by the domain scores ***S****^s^* and ***S****^t^*.


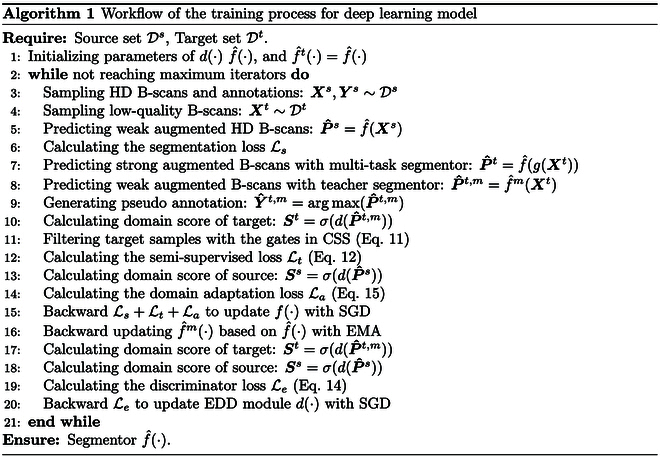


Our framework was implemented by Pytorch toolbox [46] with an Intel Core i9 CPU and an Nvidia RTX 3090 GPU. If not specially specified, the subtask segmentors were implemented by SwinUNetr [43] with feature size 24 and spatial dimensions 2, which had been respectively trained with their corresponding annotations based on the workflow of Algorithm 1. Hyperparameters {*λ*_1_, *λ*_2_, *α*, *β*_1_, *β*_2_} were set as {1.5, 0.5, 0.85, 0.1, 0.9} and {1.5, 0.5, 0.85, 0.15, 0.85} for training *f_c_*(·) and *f_v_*(·), respectively. There were a total of 250 epochs for training each subtask segmentor with an Adam optimizer [47], whose initial learning rate was 6 × 10^−4^ and was divided by 10 at 150 epochs. Batch size was set as 4 in both processes. The parameters of EDD module were updated by another Adam optimizer with a learning rate of 6 × 10^−5^.

### Experimental design and statistical analysis

To validate the segmentation performance of our framework. Thirty-nine low-quality B-scans, randomly collected from other 5 healthy subjects, were annotated and double-checked also by 3 experts to use for quantitative evaluation. The dice metric between the SV&HV segmentations ***P*** and the annotated ground-truth ***Y*** was used as the metric for evaluation (represented by SV and HV). Moreover, the performance subtask segmentors (sublayers and vessel) were also used for evaluation by the dice metric between $\hat{P}$ and $\hat{Y}$ (represented by SL, HL, and CV).

In the angiography process, the densely collected B-scans of subjects (1,024 B-scans per subject) can be directly fed into the trained multi-task segmentor (or the 2 subtask segmentors) to generate the SV&HV angiography ***Y***^**∗**^, whose mean vascular density can be directly constructed for visualizing CV distributions. Biomarker maps on the choroidal detail structure can also be computed based on ***Y***^**∗**^ to evaluate the CV distribution, including CVI, choroidal vascular index of Haller’s layer (CVI-H), and choroidal vascular index of Sattler’s layer (CVI-S).

In the statistical analysis for clinical application, evaluation metrics were expressed as mean ± SD and were compared between normal individuals and CSC patients using paired *t* test, which was performed by the Scipy package with Python. *P* value less than 0.05 was indicative of statistical significance.

## Results

### Results of ML model at segmenting choroid vessels

The proposed framework trains a machine learning (ML)-based segmentation model by specifically addressing the discrepancy between HD B-scans and low-quality B-scans. We evalutate the effectiveness of this mechanism by assessing the segmentation performance of ML model, and corresponding results are shown in Table 1. For fair comparisons, we implemented *l_s_* and *l_t_* as dice loss. First, due to the label noise of pseudo-annotations caused by the domain shift, simply using the target branch (TGB) for self-training somewhat hinders the baseline performance (1.22% lower HV dice). This issue is mitigated by adding the adaptation branch to reduce feature discrepancy, showcasing the effectiveness of modeling this task with cross-domain segmentation (4.50% improvement in HV dice). When further considering the specificities of this domain shift by our EDD module, the HV segmentation gains an additional 2.16% dice score. Finally, by incorporating our CSS module to filter pseudo-annotations with domain score, the model better considers the hard samples, resulting in further improvements in dice scores, particularly for the challenging SV segmentation (2.99% improvement).

**Table 1.** Dice scores of models for ablation studies

TGB	ADB	EDD	CSS	SL	HL	CV	SV	HV
×	×	×	×	79.05	90.55	71.59	49.98	71.66
√	×	×	×	80.41	90.89	71.30	50.88	70.44
√	√	×	×	79.66	91.91	75.02	52.76	74.94
√	√	√	×	82.78	91.91	76.80	52.98	77.10
√	√	√	√	85.46	93.27	77.28	55.97	78.47

TGB, using target branch for self-training; ADB, using adaptation branch with original discriminator; EDD, using EDD as the discriminator to discern ensembled target space; CSS, using CSS for selecting representive samples

Table 2 also investigate the effectiveness of using pseudo-annotation when supervising the target samples with *l_t_*. Our method significantly outperform the baselines by employing either soft [consistency regulation (CR) loss] or hard annotation [cross entropy (CE) loss, dice loss]. This indicates that the pseudo-annotation generated by our method contains less label noise and can effectively contribute to the training of segmentors. Notably, dice loss achieves the best performance, addressing the sample unbalanced between background and choroidal tissues. As a result, we adopt the dice loss as our *l_t_* to supervise target samples with pseudo-annotation.

**Table 2.** Dice scores of adopting different loss for our target branch

	SL	HL	CV	SV	HV
Baseline	79.05 _±0.073_	90.55 _±0.049_	71.59 _±0.022_	49.98 _±0.067_	71.66 _±0.031_
Ours + CR loss	81.23 _±0.070_	92.58 _±0.059_	75.43 _±0.038_	51.86 _±0.030_	76.53 _±0.054_
Ours + CE loss	83.80 _±0.065_	93.32 _±0.030_	75.43 _±0.036_	54.28 _±0.028_	77.91 _±0.052_
Ours + Dice loss	85.46 _±0.053_	93.27 _±0.023_	77.28 _±0.031_	55.97 _±0.052_	78.47 _±0.034_

**Fig. 4.**
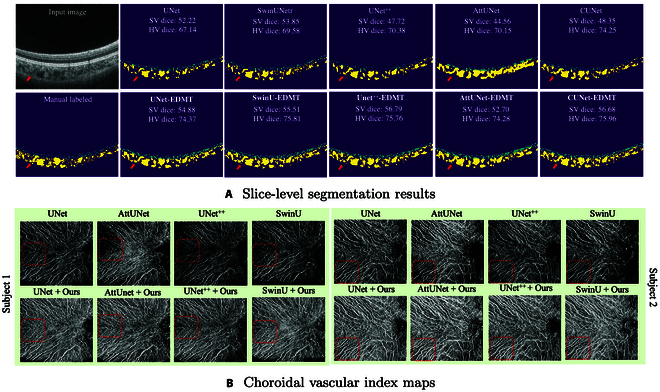
(A) Slice-level segmentation performance of our method with different backbone structures. (B) Choroidal vascular index generated by different baselines trained with or without our strategies.

We also implemented 4 backbones as the subtask segmentors to reflect the generalization property of our framework, including the CNN-based backbones UNet [41], UNet ^++^ [48], and AttUNet [42] and the multi-task backbone CUNet [23]. Figure 4A illustrates the quality of segmentations when training the segmentors with and without our proposed framework. Due to the discrepancy between the source HD B-scans and the target low-quality B-scans, the segmentors trained by the baseline methods only catch the source-like vessels, i.e., vessels have clean boundaries and regular size. Consequently, most SV and some HV located beyond the central fovea have been misclassified into the choroidal stroma, resulting in their low dice scores. Engaging our proposed framework to consider the domain discrepancy leads to better separation of CVs from choroidal stroma, contributing to higher performance. Corresponding quantitative evaluations are also given in Table 3 and Supplementary Materials. Our method improves the performance of subtask segmentors, i.e., averagely achieving 9.96% and 5.38% improvement upon baselines for SV dice and HV dice, respectively. With the more accurate slice-level segmentations, high-quality vessel biomarkers can also be generated on densely collected low-quality B-scans. As shown in Fig. 4B, our framework enhances the vascular index maps, considering more detailed structures with improved vessel consistency and more distinct vessel boundaries. Thus, our approach can better assist the computation of choroidal biomarkers in clinical practice.

**Table 3.** Dice scores of our method with different backbones

	SL	HL	CV	SV	HV
UNet	78.94 _±0.069_	91.53 _±0.038_	69.93 _±0.023_	46.74 _±0.694_	70.19 _±0.037_
UNet + Ours	84.93 _±0.058_	93.81 _±0.014_	74.73 _±0.041_	52.50 _±0.064_	77.47 _±0.035_
AttUNet	83.65 _±0.051_	93.14 _±0.028_	71.11 _±0.064_	40.20 _±0.078_	74.21 _±0.052_
AttUNet + Ours	85.79 _±0.047_	93.13 _±0.021_	76.30 _±0.035_	54.52 _±0.046_	77.09 _±0.044_
UNet^++^	83.29 _±0.054_	93.16 _±0.036_	68.71 _±0.053_	42.64 _±0.071_	70.74 _±0.060_
UNet^++^ + Ours	87.04 _±0.032_	94.65 _±0.016_	76.85 _±0.027_	57.01 _±0.036_	78.17 _±0.034_
CUNet	78.48 _±0.064_	92.24 _±0.027_	72.90 _±0.031_	45.87 _±0.056_	75.03 _±0.035_
CUNet + Ours	83.36 _±0.051_	90.06 _±0.039_	77.24 _±0.030_	55.53 _±0.054_	77.73 _±0.043_
SwinU	79.05 _±0.073_	90.55 _±0.049_	71.59 _±0.022_	49.98 _±0.067_	71.66 _±0.031_
SwinU + Ours	85.46 _±0.053_	93.27 _±0.023_	77.28 _±0.031_	55.97 _±0.052_	78.47 _±0.034_

### Results of evaluating CV distribution

**Fig. 5.**
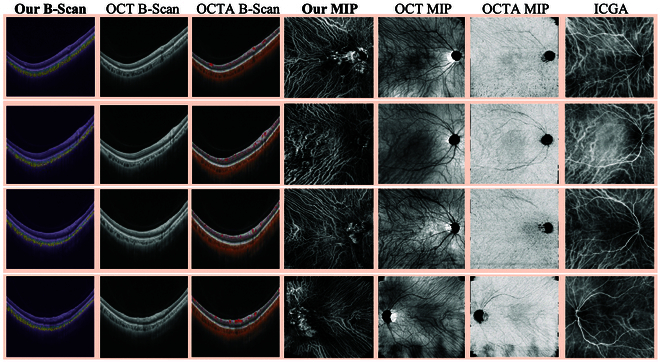
Comparison of different imaging modalities on the same subject (each row) in the slice-level (B-scan) and maximum intensity projection (MIP). Our method can efficiently extract slice-level SV&HV than OCT and OCTA. Thus, our vessel map generated by mean intensity projection of SV&HV catches more CVs as the golden standard ICGA.

**Fig. 6.**
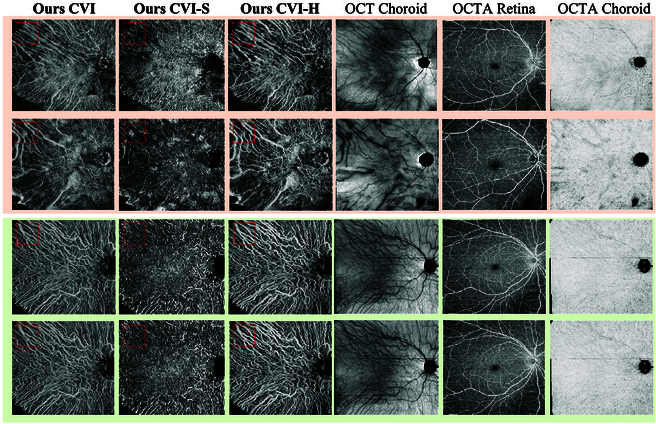
The comparison of VKH patients (apricot) and healthy subjects (green). Our method clearly reflected the abnormality of CVs on VKH patients.

Figure 5 provides the slice-level comparison between OCTA and the proposed choroidal angiography strategy. OCTA signals appear dark in the region of SV&HV due to the rapid flow of CC [12] and the melanin particles located within the RPE [14]. In contrast, our method efficiently extracts SV&HV on OCT B-scans, contributing to the better reconstruction of the CV distribution. To verify this characteristic, Fig. 6 also displays CV maps generated by the mean intensity projection of dense cube low-quality B-scans. Our method selectively reconstructs the SV&HV, reducing interferences of the choroidal stroma, capillaries, and retinal vessels. As a result, our vessel maps are less affected by artifacts compared to OCT/OCTA en face.

We also collected the ICGA for comparison, which serves as the golden standard for imaging CVs. As shown in Fig. 5, our vessel maps exhibit a similar distribution to ICGA, indicating that the CVs are well captured by our framework. Additionally, our choroidal angiography strategy is noninvasive and does not affect the metabolism of dyes as ICGA, making our vessel maps to have more clear distribution. Moreover, our strategy can also capture the slice-level vessels for different choroidal sublayers, supporting the computation of additional biomarkers such as CVI-H, CVI-S, choroidal thickness of HL (CT-H), and choroidal thickness of SL (CT-S).

### Results of clinical applications

Although trained exclusively on B-scans collected from healthy subjects, our method demonstrates versatility in reconstructing the CV for patients with choroidal abnormal. Figure 6 illustrates the comparison between healthy subjects and patients with chronic VKH disease, characterized by inflammation in the choroid [4]. Remarkably, our method accurately reconstructs SV&HV structure even for patients with abnormal choroidal structures. Additionally, the high-resolution biomarker maps computed by our methods also vividly highlight the abnormal distribution of CVs in VKH patients [49], which cannot be captured by other noninvasive imaging modalities. These results underscore the clinical potential of our method, making it a promising tool for screening various choroidal diseases.

**Fig. 7.**
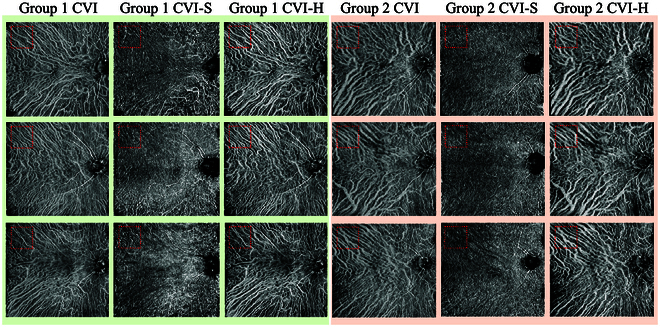
Visualization of the choroidal indexes for examples in the healthy (green, group 1) and CSC (apricot, group 2) groups. Choroidal vessels shrink at the far end of macular fovea for the CSC group.

In addition to screening abnormal CV distribution, our proposed method offers quantative analysis capabilities for patients with choroidal disease. Table 4 presents the CV biomarkers (CVI, CVI-H, CVI-S) within the circular regions of varying radii from the central macular fovea. The results indicate that the choroidal vascular indexes of the CSC group are lower than those of healthy group (2.9%, 3.2%, and 3.0% on average for CVI, CVI-H, and CVI-S, respectively). Significant differences (*P* < 0.05) are observed between these 2 groups, particularly in the regions farther from the fovea (6-, 9-, and 12-mm radiuses). Figure 7 visually represents the choroidal vascular indexes of examples from both groups.

**Table 4.** Layer-wise choroidal vascular indexes between healthy and CSC groups. Group 1: healthy group; Group 2: CSC group; bold style: statistically significant *P*.

	CVI			CVI-H			CVI-S		
Region	Group 1	Group 2	*P*	Group 1	Group 2	*P*	Group 1	Group 2	*P*
1 mm	0.482 _±0.041_	0.466 _±0.034_	0.149	0.422 _±0.057_	0.409 _±0.079_	0.428	0.407 _±0.088_	0.379 _±0.101_	0.259
3 mm	0.481 _±0.034_	0.461 _±0.035_	**0.028**	0.411 _±0.046_	0.391 _±0.070_	0.128	0.415 _±0.056_	0.383 _±0.027_	**0.028**
6 mm	0.490 _±0.029_	0.463 _±0.032_	**<0.001**	0.436 _±0.036_	0.390 _±0.070_	**<0.001**	0.431 _±0.047_	0.399 _±0.037_	**0.013**
9 mm	0.488 _±0.026_	0.451 _±0.034_	**<0.001**	0.461 _±0.031_	0.420 _±0.067_	**<0.001**	0.440 _±0.043_	0.405 _±0.031_	**0.002**
12 mm	0.480 _±0.023_	0.441 _±0.038_	**<0.001**	0.473 _±0.029_	0.433 _±0.067_	**<0.001**	0.444 _±0.038_	0.400 _±0.031_	**<0.001**

## Discussion

### Summary of main results

The proposed framework significantly enhances ML-based segmentation for medical imaging by addressing the discrepancies between HD B-scans and original low-quality B-scans. This innovation brings notable improvements in both performance and clinical applicability. Specifically, introducing an adaptation branch to minimize feature discrepancies led to a 4.50% improvement in HV dice scores. Further refinement with the EDD module added another 2.16%, while the CSS module boosts SV dice by 2.99%. Additionally, this robust approach was validated across 4 different backbones—UNet, UNet++, AttUNet, and CUNet—showing an average improvement of 9.96% in SV dice and 5.38% in HV dice.

Clinically, the framework excels in generating high-quality CV maps that are comparable to the gold standard ICGA and superior to OCTA. It efficiently extracts SV and HV on OCT B-scans, avoiding the need for invasive dyes and providing clearer vessel distribution. The framework also demonstrated its versatility by accurately reconstructing CVs in patients with VKH disease, vividly highlighting abnormal distributions. Additionally, the framework offers quantitative analysis capabilities for choroidal diseases. In a study comparing healthy individuals with CSC patients, it revealed significantly lower choroidal vascular indexes in the CSC group, particularly in regions farther from the fovea. We believe that the proposed framework can provide valuable insights when exploring abnormalities in CVs, making it a promising tool for retinal disease analysis.

### Influence of the sample’s number

**Fig. 8.**
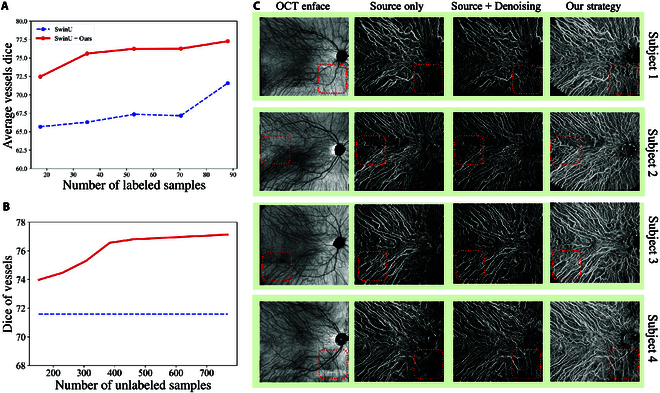
(A) Adopting different number of unannotated low-quality B-scans with the 88 annotated HD B-scans for training with our proposed strategy (red line) or baseline (blue line). (B) Adopting different number of annotated HD B-scans with the 768 unannotated low-quality B-scans for training with our proposed strategy (red line) or baseline (blue line). (C) Comparison between the source-trained segmentation model, simply adopting image denoising method before segmentation, and the proposed cross-domain segmentation strategy.

The proposed strategy adopts unlabeled low-quality B-scan to assist the training of the segmentation model under insufficient annotated HD B-scan [22,23]. Here, we explore the influence of the number of labeled samples and unlabeled samples for training with our strategy. As indicated in Fig. 8A, our strategy can observably improve the vessel segmentation performance, especially when the annotated samples are fewer. With the proposed strategy, the vessel dice can achieve 72.47 when just using 18 HD B-scans for training, which is even higher than the performance of training with nearly 5 times annotated samples (88 HD B-scans) in a fully supervised manner. In addition, the influence of the number of unlabeled low-quality B-scan is also explored in Fig. 8B, indicating that adopting 400 unlabeled low-quality B-scan for the proposed strategy is sufficient to achieve a satisfactory performance (76.25 dice score).

### Denoising or adaptation

The domain shift between HD B-scans and low-quality B-scans is partially caused by their different imaging quality, as discussed in Introduction and Results. Here, we compare the proposed cross-domain segmentation strategy with simply adopting denoising methods [50] for assistance. Corresponding results are given in Fig 8C. Although the speckle noise can be reduced, the feature discrepancy caused by the imaging setting will also be stretched out by the denoising step. Thus, simply denoising before segmentation even weaken the discernment of indistinct vessels and aggravates the vessel inconsistency. Compared with it, our cross-domain segmentation strategy can better deal with both the influence of speckle noise and the imaging settings, contributing to our better reconstruction performance for detailed vessel structures.

### Comparison with other DA and SSL strategies

The core of our framework is to consider more about the specificities of the discrepancy between HD B-scans and low-quality B-scans. To better show the effectiveness of this part, we also implemented 9 SSL and DA methods for comparison, including ST [25], CPS [26] FM [30], CCT [28], MT [29], ASOS [34], CFEA [32], MDDA [33], and DACS [37].

**Fig. 9.**
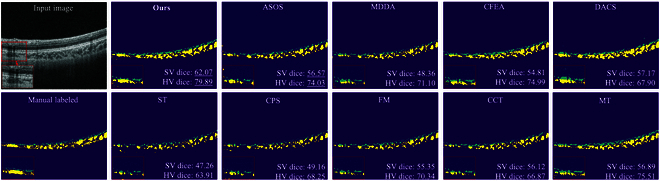
Comparison of the segmentation performance on low-quality B-scans between the proposed methods and other SSL and DA methods.

Figure 9 visualizes the results of our work and other SSL&DA methods. It can be seen that the SSL strategies with hard pseudo-annotations (ST, FM, and CPS) tend to overfit the source domain, classifying a large number of vessels as stroma. This is because those methods use the source-trained segmentor to generate pseudo-annotations without concerning the different distribution between HD B-scans and low-quality B-scans, making the pseudo-annotation suffer from large label noise. Although utilizing soft supervision (CCT and MT) somewhat solves the distribution mismatch between the labeled and unlabeled images, it cannot effectively handle the hard samples [51]. Compared with them, our method can reduce the label noise by adopting the proposed CCS module to filter samples based on the domain score **S**, which contributes to our higher performance.

Compared with the SSL methods, DA strategies usually have better performance. This verifies the rationality of our framework to model the SV&HV reconstruction as a DA task. However, some settings of existing DA methods are still unsuitable for our case. Specifically, the augmentation-based method DACS mixes target objects into the source images to form the mixed domain samples. However, in our case, this may violate the structure constraint, for example, SV must locate on the top of HV, causing the structure errors in Fig. 9. In addition, due to the different imaging quality between source HD B-scans and target low-quality B-scans, other adversarial learning based methods easily discern the speckle noise as the domain discriminative cues. Thus, these methods cannot efficiently consider the distribution shift of the choroidal structure, making them still overfit the source, i.e., MDDA, or suffer from noise boundaries, i.e.*,* ASOS and CFDA. Compared with them, our EDD module solves this by discerning the temporally ensembled output space of low-quality B-scans, which simulated the imaging process of HD B-scans to reduce the influence of quality gap.

**Fig. 10.**
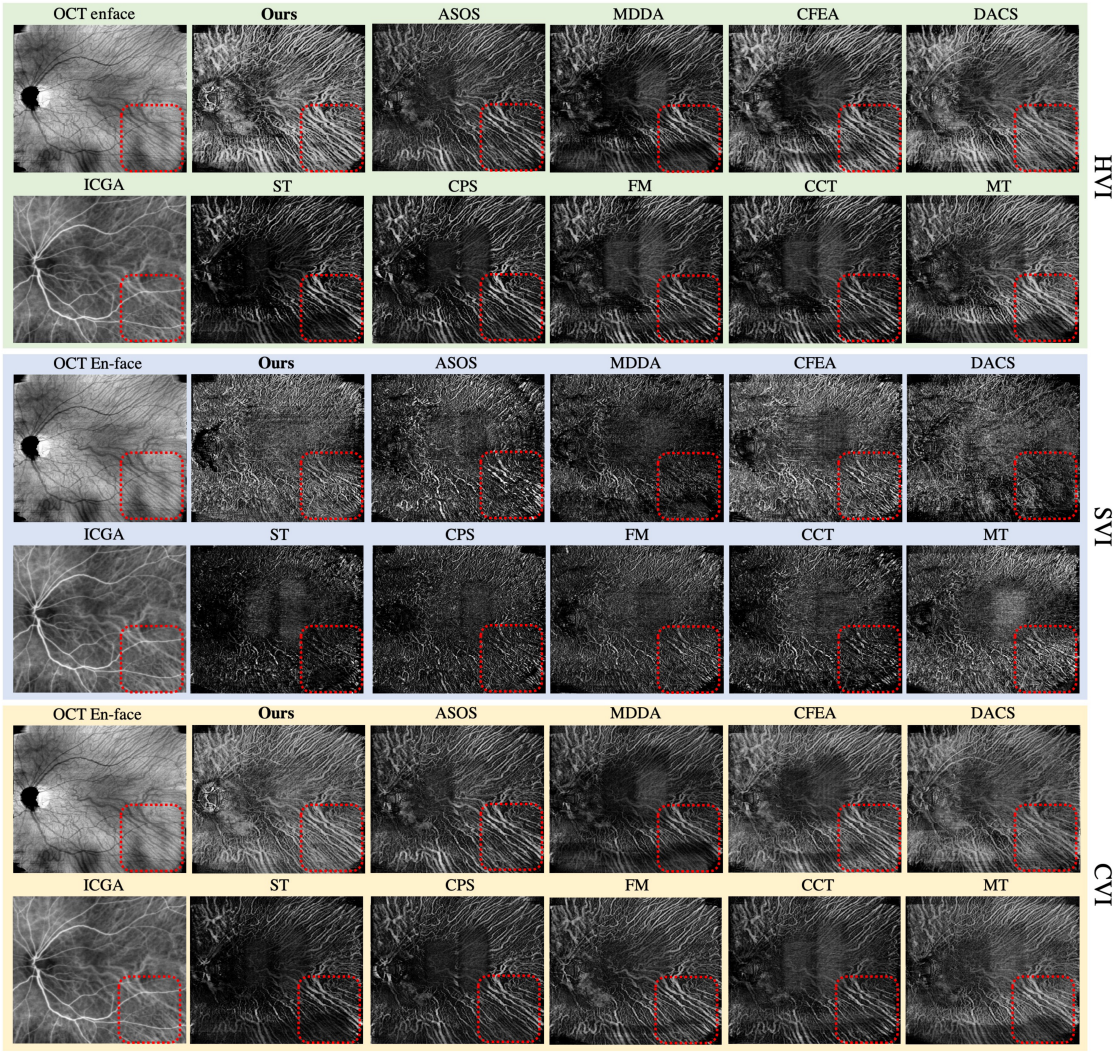
Comparison of the choroidal vascular index maps between our method and other methods.

Table 5 also quantitatively evaluates those methods in SV&HV segmentation. It can be seen that our method achieves the best performance on all CV metrics, indicating our superior efficiency in SV&HV extraction. Only the augmentation-based method DACS outperforms our method in SL dice. This is because the augmentation strategy of DACS can solve the sample insufficiency of SL&SV. However, as discussed above, this also causes structure errors, which limits its angiography capability and potential clinic applications. Finally, Fig. 10 also gives the results of the different methods for the proposed choroidal angiography. High-qulity choroidal vascular index map can be computed by angiography with our framework due to the better slice-level segmentations and the consideration of the specificities of this task.

**Table 5.** Comparison with other SSL and DA mechanisms on dice scores

	SL	HL	CV	SV	HV
SwinU	79.05	90.55	71.59	49.98	71.66
SwinU + ST	78.44	90.57	65.48	45.48	64.77
SwinU + CPS	82.75	92.50	67.70	47.02	69.28
SwinU + FM	84.13	92.13	71.30	51.21	70.74
SwinU + CCT	82.79	92.26	71.89	50.99	72.74
SwinU + MT	82.64	92.16	75.33	54.03	75.51
SwinU + ASOS	76.28	90.53	72.84	51.23	72.61
SwinU + MDDA	80.24	92.02	71.10	48.48	71.53
SwinU + CFEA	80.59	91.26	74.81	52.86	75.47
SwinU + DACS	87.29	92.18	74.70	53.98	77.69
SwinU + Ours	85.46	93.27	77.28	55.97	78.47

### Limitations

Although our work provides an effective tool for choroidal angiography to evaluate vessel distribution of choroidal sublayers, some limitations are still unresolved and should be explored in future works. First, our framework cannot be utilized to catch capillary in CC because its volume in OCT B-scans is too low to discern by the expert. This also makes our method not better at distinguishing some SV from capillary, rising some noise on our SV density map, especially near the macula. Considering the effectiveness of OCTA on catching capillary [1], we think that using sequential OCT signals to consider the blood flow may solve this problem to a certain extent. Second, the shadow artifact is another problem of our framework. As shown in Fig. 5, although our method has less shadow artifact than other imaging modalities, some retinal shadows still exist near the optic disk. In addition, our CVI-S map still suffers from retinal shadow and even the shadow of HV, because the HV influences the lower bound of SL. Engaging the shadow elimination method [19] may somewhat alleviate this limitation. The last concern is the computational cost. Specifically, using our framework for training requires additional computational resources for the target branch and adaptation branch, which cost 16.80G and 24.08G floating-point operations per second (FLOPs), respectively. Although the reconstruction process does not incur these additional costs, our framework still needs to perform SV and HV segmentation on 1,024 B-scans to compute the choroidal biomarkers. We hope that future work can simplify this process to enhance the speed and efficiency.

## Conclusion

We propose a choroidal angiography to visualize and evaluate vessels of choroidal sublayers. Our approach focuses on segmenting the SV&HV from densely collected OCT B-scans to reconstruct the CV distribution, and considers the gap between the HD B-scans and the low-quality B-scans. A deep learning-based framework is proposed to utilize additional unlabeled samples for self-training, which adopts the CSS and EDD module to reduce the label noise of pseudo-annotations and better engage adversarial training, respectively. Experiments show the effectiveness of the proposed angiography, as well as demonstrate the clinical potential in choroid analysis, observing that patients with CSC have significantly (*P* < 0.05) lower vascular indexes at all choroidal sublayers than healthy individuals, especially in the region beyond central fovea of macula (larger than 6 mm).

## Data Availability

Training and inference codes of this work will be released at https://github.com/zh460045050/COCTA. The dataset will be uploaded at https://wiki.milab.wiki/display/LF/Open+Source+Project after approval.

## Supplementary Materials

Supplementary 1 A glossary is provided in Table 6 to clarify the definition of some pivotal notations. Tables 7 and 8 report additional recall scores and intersection over union (IoU), which are in accordance with the dice score, indicating the effectiveness of our framework
