# Supplementary material for: Choroidal Optical Coherence Tomography Angiography: Noninvasive Choroidal Vessel Analysis via Deep Learning
Source: Health Data Sci. 2024 Sep 10;4:0170. doi: 10.34133/hds.0170 (PMC11383389; doi:10.34133/hds.0170)
Supplement: Supplementary 1 — A glossary is provided in Table 6 to clarify the definition of some pivotal notations. Tables 7 and 8 report additional recall scores and intersection over union (IoU), which are in accordance with the dice score, indicating the effectiveness of our framework [file hds.0170.f1.docx]

### Supplementary Materials

A glossary is provided in Table 6 to clarify the definition of some pivotal notations. Table 7 and Table 8 report additional recall scores and intersection over union (IoU), which are in accordance with the dice score, indicating the effectiveness of our framework.

Table 6: Definition of Some Pivotal Notions

| $\mathbf{X}\in\mathbb{R}^{T\times H\times W}$ | the cube of B-scans. |
| --- | --- |
| $\mathbf{Y}\in\mathbb{R}^{T\times H\times W}$ | the label volume, indicating the choroidal sublayer vessels. |
| $\mathbf{Y}^{*}\in\mathbb{R}^{T\times H\times W}$ | the choroidal sublayer vessel prediction of $\mathbf{X}$. |
| $\mathbf{P}\in\mathbb{R}^{3\times T\times H\times W}$ | the choroidal sublayer vessel probability of $\mathbf{X}$. |
| $\mathbf{X}^{s}\in\mathbb{R}^{H\times W}$ | the source HD B-scan. |
| $\mathbf{X}^{t}\in\mathbb{R}^{H\times W}$ | the target original low-quality B-scan. |
| $\mathbf{Y}\in\mathbb{R}^{H\times W}$ | the choroidal sublayer vessel prediction of a B-scan. |
| $\mathbf{Y}^{s,\_}\in\mathbb{R}^{\_\times H\times W}$ | the prediction of source HD B-scan. |
| $\mathbf{Y}^{t,\_}\in\mathbb{R}^{\_\times H\times W}$ | the prediction of target B-scan. |
| $\mathbf{Y}^{\_,l}\in\mathbb{R}^{H\times W}$ | the choroidal sublayer prediction. |
| $\mathbf{Y}^{\_,v}\in\mathbb{R}^{H\times W}$ | the choroidal vessel prediction. |
| $\mathbf{Y}^{\_,m}\in\mathbb{R}^{H\times W}$ | the prediction of teacher segmentor. |
| $\mathbf{P}\in\mathbb{R}^{H\times W}$ | the choroidal sublayer vessel probability of a B-scan. |
| $\mathbf{P}^{s,\_}\in\mathbb{R}^{\_\times H\times W}$ | the prediction of source HD B-scan. |
| $\mathbf{P}^{t,\_}\in\mathbb{R}^{\_\times H\times W}$ | the probability of target B-scan. |
| $\mathbf{P}^{\_,l}\in\mathbb{R}^{3\times H\times W}$ | the choroidal sublayer probability. |
| $\mathbf{P}^{\_,v}\in\mathbb{R}^{2\times H\times W}$ | the choroidal vessel probability. |
| $\mathbf{P}^{\_,m}\in\mathbb{R}^{\_\times H\times W}$ | the probability outputed by teacher segmentor. |
| $\mathbf{M}^{c}\in\mathbb{R}^{H\times W}$ | the confident gate. |
| $\mathbf{M}^{d}\in\mathbb{R}^{H\times W}$ | the domain gate. |
| $\mathbf{S}\in\mathbb{R}^{H\times W}$ | the distribution probability. |
| $T$ | the total number of low-quality B-scans in the cube. |
| $H$ | the total hight of each B-scan. |
| $W$ | the total width of each B-scan. |
| $N^{t}$ | the number of targe samples (low-quality B-scans). |
| $\lambda_{1}$ | the weight to contral the self-training loss. |
| $\lambda_{2}$ | the weight to contral the adaptation loss. |
| $\alpha$ | the threshold to contral the confident gate. |
| $\beta_{1}$ | the lower bound of the domain gate. |
| $\beta_{2}$ | the upper bound of the domain gate. |
| $\mathcal{D}^{s}$ | The source sample set that contains the label and image of HD B-scans. |
| $\mathcal{D}^{t}$ | The target sample set that contains the image of original (low-quality) B-scans. |
| $\mathcal{L}_{s}(\mathcal{D}^{s})$ | The segmentation loss. |
| $\mathcal{L}_{t}(\mathcal{D}^{t})$ | The self-training loss. |
| $\mathcal{L}_{a}(\mathcal{D}^{s},\mathcal{D}^{t})$ | The adaptation loss. |
| $\mathcal{L}_{e}(\mathcal{D}^{s},\mathcal{D}^{t})$ | The discriminator loss. |
| $f(\cdot):\mathbb{R}^{H\times W}\to\mathbb{R}^{3\times H\times W}$ | The function of sub-task segmentors. |
| $f(\cdot):\mathbb{R}^{H\times W}\to\mathbb{R}^{3\times H\times W}$ | The function of teacher segmentor. |
| $\hat{f}(\cdot):\mathbb{R}^{H\times W}\to\mathbb{R}^{3\times H\times W}$ | The function of multi-task segmentor. |
| $f_{l}(\cdot):\mathbb{R}^{H\times W}\to\mathbb{R}^{3\times H\times W}$ | The function of choroidal sublayer segmentor. |
| $f_{v}(\cdot):\mathbb{R}^{H\times W}\to\mathbb{R}^{2\times H\times W}$ | The function of choroidal vessel segmentor. |
| $d(\cdot):\mathbb{R}^{H\times W}\to\mathbb{R}^{H\times W}$ | The function of the EDD module. |
| $\sigma(\cdot):\mathbb{R}^{H\times W}\to\mathbb{R}^{H\times W}$ | The sigmoid operation. |
| $l_{t}(\cdot):\mathbb{R}^{H\times W}\to\mathbb{R}^{1}$ | The function of dice loss. |

Table 7: Recall of our method with different backbones

|  | SL-Recall | HL-Recall | CV-Recall | **SV-Recall** | **HV-Recall** |
| --- | --- | --- | --- | --- | --- |
| UNet | 79.01${}_{\pm0.048}$ | 88.72${}_{\pm0.053}$ | 61.59${}_{\pm0.043}$ | 44.13${}_{\pm0.083}$ | 59.54${}_{\pm0.055}$ |
| UNet + Ours | 84.55${}_{\pm0.036}$ | 91.31${}_{\pm0.032}$ | 78.30${}_{\pm0.035}$ | 59.79${}_{\pm0.075}$ | 77.72${}_{\pm0.046}$ |
| AttUNet | 82.77${}_{\pm0.043}$ | 90.63${}_{\pm0.046}$ | 69.06${}_{\pm0.079}$ | 35.82${}_{\pm0.081}$ | 73.08${}_{\pm0.081}$ |
| AttUNet + Ours | 82.87${}_{\pm0.053}$ | 91.44${}_{\pm0.274}$ | 82.85${}_{\pm0.043}$ | 63.13${}_{\pm0.062}$ | 81.71${}_{\pm0.052}$ |
| UNet${}^{++}$ | 80.52${}_{\pm0.059}$ | 93.42${}_{\pm0.029}$ | 58.65${}_{\pm0.079}$ | 33.53${}_{\pm0.077}$ | 61.89${}_{\pm0.089}$ |
| UNet${}^{++}$ + Ours | 84.35${}_{\pm0.038}$ | 94.58${}_{\pm0.024}$ | 81.47${}_{\pm0.039}$ | 68.41${}_{\pm0.065}$ | 79.84${}_{\pm0.044}$ |
| CUNet | 75.76${}_{\pm0.065}$ | 90.47${}_{\pm0.034}$ | 65.42${}_{\pm0.050}$ | 38.95${}_{\pm0.062}$ | 68.51${}_{\pm0.053}$ |
| CUNet + Ours | 87.18${}_{\pm0.039}$ | 92.09${}_{\pm0.039}$ | 81.07${}_{\pm0.037}$ | 70.43${}_{\pm0.044}$ | 77.10${}_{\pm0.053}$ |
| SwinU | 80.05${}_{\pm0.047}$ | 90.16${}_{\pm0.036}$ | 62.73${}_{\pm0.046}$ | 47.71${}_{\pm0.082}$ | 61.07${}_{\pm0.044}$ |
| SwinU + Ours | 86.98${}_{\pm0.036}$ | 91.02${}_{\pm0.031}$ | 83.60${}_{\pm0.038}$ | 71.10${}_{\pm0.063}$ | 80.45${}_{\pm0.039}$ |

Table 8: IoU of our method with different backbones

|  | SL-IoU | HL-IoU | CV-IoU | **SV-IoU** | **HV-IoU** |
| --- | --- | --- | --- | --- | --- |
| UNet | 65.74${}_{\pm0.094}$ | 84.62${}_{\pm0.064}$ | 53.81${}_{\pm0.027}$ | 30.76${}_{\pm0.057}$ | 54.20${}_{\pm0.044}$ |
| UNet + Ours | 74.23${}_{\pm0.083}$ | 88.38${}_{\pm0.025}$ | 59.82${}_{\pm0.051}$ | 35.85${}_{\pm0.057}$ | 63.36${}_{\pm0.047}$ |
| AttUNet | 72.21${}_{\pm0.072}$ | 87.30${}_{\pm0.047}$ | 55.54${}_{\pm0.074}$ | 25.46${}_{\pm0.062}$ | 59.27${}_{\pm0.066}$ |
| AttUNet + Ours | 75.41${}_{\pm0.069}$ | 87.21${}_{\pm0.037}$ | 61.81${}_{\pm0.046}$ | 37.61${}_{\pm0.043}$ | 62.93${}_{\pm0.059}$ |
| UNet${}^{++}$ | 71.73${}_{\pm0.077}$ | 87.39${}_{\pm0.060}$ | 52.59${}_{\pm0.060}$ | 27.36${}_{\pm0.056}$ | 55.04${}_{\pm0.069}$ |
| UNet${}^{++}$ + Ours | 77.19${}_{\pm0.049}$ | 89.88${}_{\pm0.029}$ | 62.49${}_{\pm0.036}$ | 39.96${}_{\pm0.035}$ | 64.30${}_{\pm0.046}$ |
| CUNet | 65.03${}_{\pm0.084}$ | 85.71${}_{\pm0.045}$ | 57.45${}_{\pm0.038}$ | 29.93${}_{\pm0.046}$ | 60.16${}_{\pm0.046}$ |
| CUNet + Ours | 71.79${}_{\pm0.073}$ | 82.15${}_{\pm0.063}$ | 63.02${}_{\pm0.040}$ | 38.64${}_{\pm0.051}$ | 63.77${}_{\pm0.058}$ |
| SwinU | 65.96${}_{\pm0.098}$ | 83.09${}_{\pm0.078}$ | 55.81${}_{\pm0.027}$ | 33.58${}_{\pm0.058}$ | 55.93${}_{\pm0.038}$ |
| SwinU + Ours | 74.98${}_{\pm0.079}$ | 87.48${}_{\pm0.039}$ | 63.07${}_{\pm0.040}$ | 39.04${}_{\pm0.049}$ | 64.70${}_{\pm0.046}$ |
